# Supplementary material for: A Regionally Determined Climate‐Informed West Nile Virus Forecast Technique
Source: Geohealth. 2026 Jan 29;10(2):e2025GH001657. doi: 10.1029/2025GH001657 (PMC12856058; doi:10.1029/2025GH001657)
Supplement: Supplementary file 1 — Supporting Information S1 [file GH2-10-e2025GH001657-s001.pdf]

Supporting Information: A Regionally Determined Climate-Informed West Nile Virus Forecast Technique

Ryan D. Harp[1,2,3,4,5,*], Karen M. Holcomb[2], Stanley G. Benjamin[1,6], Benjamin W. Green[1,6], Hunter Jones[7], and Michael A. Johansson[8,9]

[1] Global Systems Laboratory, National Oceanic and Atmospheric Administration; Boulder, CO, USA
[2] Division of Vector-Borne Diseases, Centers for Disease Control and Prevention; Fort Collins, CO, USA
[3] Cooperative Programs for the Advancement of Earth System Science, University Corporation for Atmospheric Research; Boulder, CO, USA
[4] Physical Sciences Laboratory, National Oceanic and Atmospheric Administration; Boulder, CO, USA
[5] Climate Adaptation Partnership, University of Minnesota; St. Paul, MN, USA
[6] Cooperative Institute for Research in Environmental Science, University of Colorado Boulder; Boulder, CO, USA
[7] Climate Program Office, National Oceanic and Atmospheric Administration; Silver Spring, MD, USA
[8] Division of Vector-Borne Diseases, Centers for Disease Control and Prevention; San Juan, PR, USA
[9] Network Science Institute and Bouvé College of Health Sciences, Northeastern University; Boston, MA, USA

[*] Corresponding author: Ryan D. Harp (ryanharp@umn.edu)

Contents of this file

Text S1–S3
Figures S1–S24
Tables S1–S7

Text S1: Mathematical Description of Weighted Interval Score

We used a weighted interval score (WIS) to assess the skill of our forecast and comparison models. As described in Bracher et al. (2021), the WIS for quantile forecasts *F* and observed cases *O* can be readily calculated as:

$$WIS_{\alpha_{\{0:k\}}}(F,\ O) = \frac{1}{K+1/2} \times (w_0 \times |O - m| + \sum_{k=1}^{K}\{w_k \times IS_{\alpha_k}(F, O)\})$$

for a set of $k = 1, \ldots, K$ prediction intervals, weights $w_k = \frac{\alpha_k}{2}$ (w_0 simplifies to $\frac{1}{2}$), α is the difference between 1 and the prediction interval (i.e., an 80% prediction interval–described by the 10th percentile and 90th percentile quantiles–leads to a corresponding α value of 0.2 [1 - 0.8]), and the median *m*, where the interval score (IS) is calculated as:

$$IS = (q_{upper} - q_{lower}) + \ldots \qquad \text{(dispersion term)}$$
$$\frac{2}{\alpha}(q_{lower} - O) \times 1(O < q_{lower}) + \ldots \qquad \text{(overprediction term)}$$
$$\frac{2}{\alpha}(O - q_{upper}) \times 1(O > q_{upper}) \qquad \text{(underprediction term)}$$

where q_{upper} and q_{lower} are the forecast values at the upper and lower quantiles, and 1 is an indicator function that activates the appropriate under- or overprediction term if the observed value is below q_{lower} or above q_{upper}.

Prior to scoring our forecast and comparison model, we first log-transformed the forecast quantiles and observations to reduce a known bias in WIS for higher-value forecasts:

$$F'_{q,\,region,\,year} = ln(F_{q,\,region,\,year} + 1)$$

$$O'_{q,\,region,\,year} = ln(O_{q,\,region,\,year} + 1)$$

where *F′* are the log-transformed forecasts and *O′* are the log-transformed observed WNND cases. Model scoring was subsequently based on *F′* and *O′*.

Text S2: Descriptions of Model Development

We used a generalized linear Bayesian regression model to produce probabilistic climate-informed regional-level forecasts of annual WNND cases. This was performed for both

univariable and bivariable climate-informed forecasts to determine the influence of X_i regional climate variables on annual WNND cases. These regressions were fit using the stan_glm.nb function in the *rstanarm* package in *R* (Goodrich et al., 2023; R Core Team, 2023) and are of the following form:

$$cases \sim Negative\ Binomial(\mu_i, \phi)$$
$$\mu_i = \beta_0 + \beta_1 X_1 \text{ (univariable form) or}$$
$$\mu_i = \beta_0 + \beta_1 X_1 + \beta_2 X_2 \text{ (bivariable form)}$$

where μ is the mean, ϕ is the dispersion, β_0 is the intercept, and β_1 and β_2 are fit regression coefficients for the selected climate covariate. We ran four chains for each regression with a burn-in of 500 samples, then collected an additional 500 for each chain, resulting in 2,000 total samples across the four chains.

We used a similar generalized linear mixed-effects regression model in a Bayesian framework to produce probabilistic climate-informed county-level forecasts of annual WNND cases driven by regional-level climate conditions. This was also performed for both univariable and bivariable climate-informed forecasts to determine the influence of X_i regional climate variables on county-level annual WNND cases, with county as a random effect. These regressions were fit using the stan_glmer.nb function in the *rstanarm* package in *R* (Goodrich et al., 2023; R Core Team, 2023) and had the following form:

$$cases \sim Negative\ Binomial(\mu_i, \phi)$$
$$\mu_i = \beta_0 + \beta_1 X_1 + (1 \mid county) \text{ (univariable form) or}$$
$$\mu_i = \beta_0 + \beta_1 X_1 + \beta_2 X_2 + (1 \mid county) \text{ (bivariable form)}$$

where μ is the mean, ϕ is the dispersion, β_0 is the intercept, and β_1 and β_2 are regression coefficients. We ran four chains for each regression with a burn-in of 500 samples, then collected an additional 500 for each chain, resulting in 2,000 total samples across the four chains.

Text S3: Description of Regional Aggregation of Ensemble Forecasts

We used two approaches to produce regional benchmark forecasts from county-level ensemble forecasts from the 2022 West Nile Virus Forecasting Challenge. First, we performed a spatially dependent approach where the *q*-quantile forecast for a given region is equal to the sum of the *q*-quantile forecast values from all counties within that region:

$$forecast_{region,\, q} \; = \; \sum_{i=1}^{c} forecast_{i,\, q}$$

where c is the set of all counties in a region and q is the set of 23 quantiles in {0.01, 0.025, 0.05, 0.10, …, 0.90, 0.95, 0.975, 0.99}.

We also applied a spatially independent approach where the q-quantile forecast for a given region was generated from a distribution of the sum of counts sampled randomly across each county in each region (n = 1,000). Each bootstrapped regional forecast is generated by adding one random sample from each c county forecast within the region. We denote $forecast^*_{region}$ as a sample regional forecast determined from a single bootstrapped sample across all c counties in the region:

$$forecast\ ^*_{region} = \sum_{i=1}^{c} N_i$$

such that N_i is a random sample from the county-specific distribution of case forecasts. The q-quantile forecast using n = 1,000 bootstrapped samples for that region is then:

$$forecast_{region,\, q} \; = \; Q\left(forecast\ ^*_{region,\, 1\ldots n},\ q\right)$$

where Q is the quantile at $q\ in\ \{0.01,\ 0.025,\ 0.05,\ 0.10,\ ..\ ,\ 0.90,\ 0.95,\ 0.975,\ 0.99\}$.

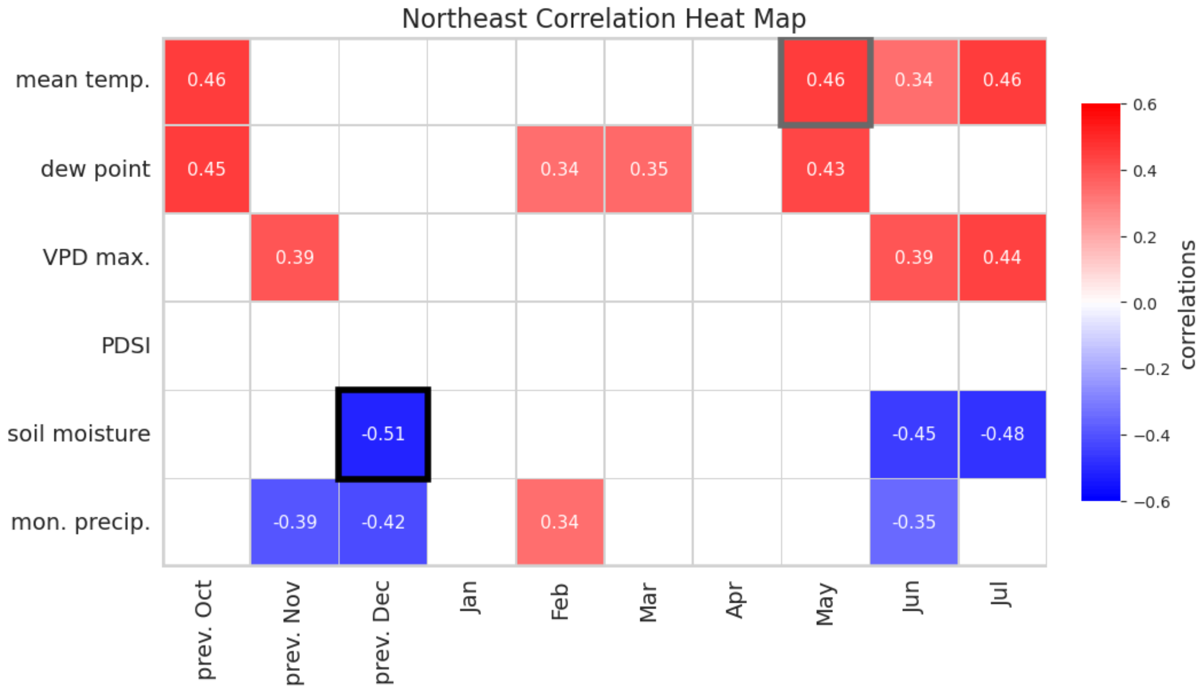

Figure S1: Correlations between Monthly Climate Factors and Annual West Nile Virus Neuroinvasive Disease in the Northeast NEON Region. Each square shows the Pearson correlation (annotated red-blue fill) between annual WNND and the monthly climate factor represented by the given square for the Northeast NEON region. For example, the top right square represents the relationship between July mean temperature and annual WNND of the concurrent year (note that October–December columns represent correlations between climate conditions of the previous year with annual WNND as concurrent year climate conditions would have minimal impact on annual WNND totals given the strong August–September peak in WNV). Correlations between ±0.32 (*p*-value ~0.2) are masked to highlight stronger climate-WNND relationships. The regional primary and secondary climate variables selected for the bivariable WNV forecast model are outlined in thick black and gray, respectively.

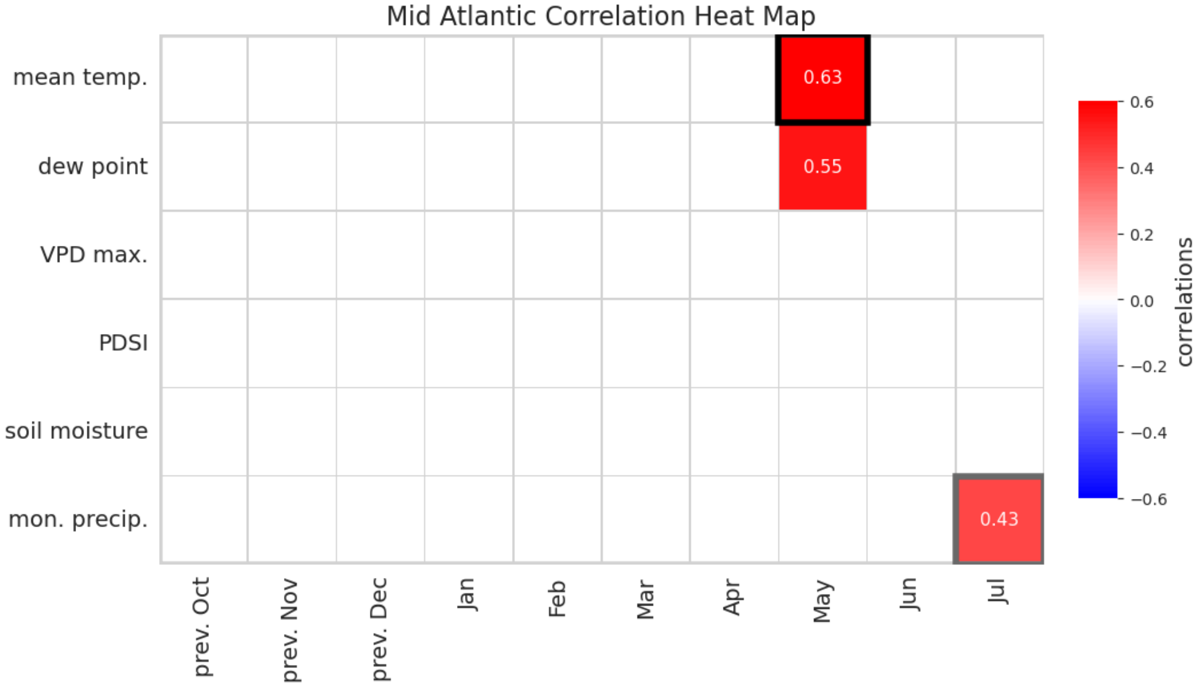

Figure S2: Correlations between Monthly Climate Factors and Annual West Nile Virus Neuroinvasive Disease in the Mid Atlantic NEON Region. Same as Figure S1 but for the Mid Atlantic NEON region.

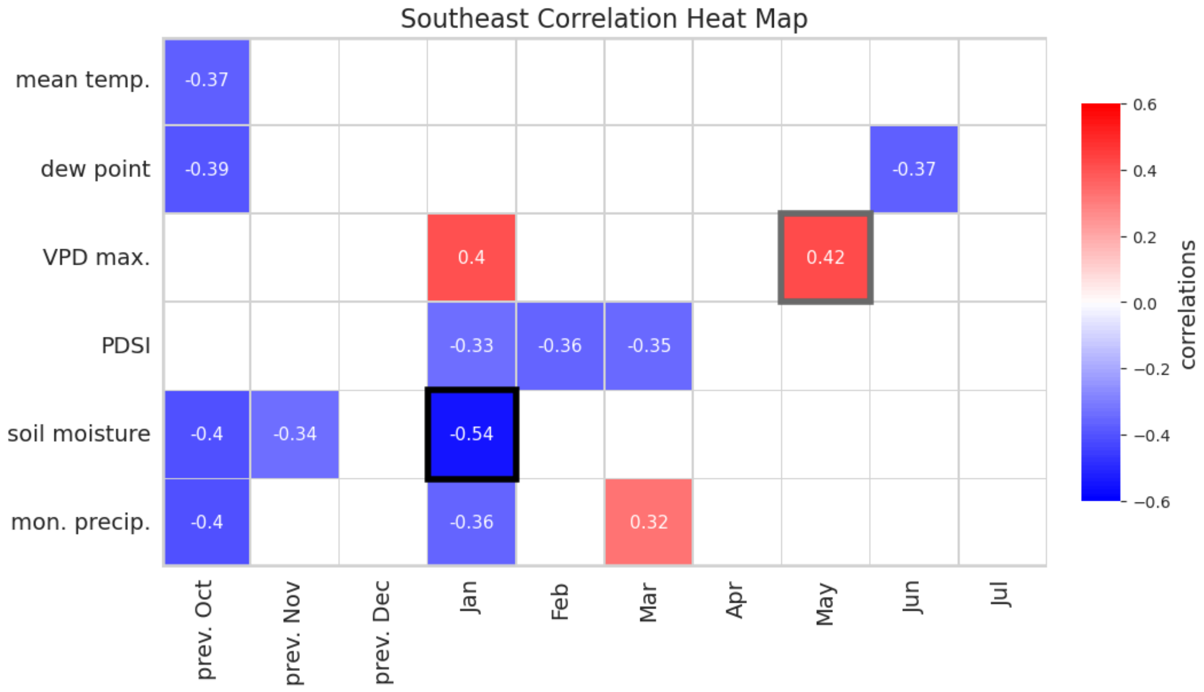

Figure S3: Correlations between Monthly Climate Factors and Annual West Nile Virus Neuroinvasive Disease in the Southeast NEON Region. Same as Figure S1 but for the Southeast NEON region.

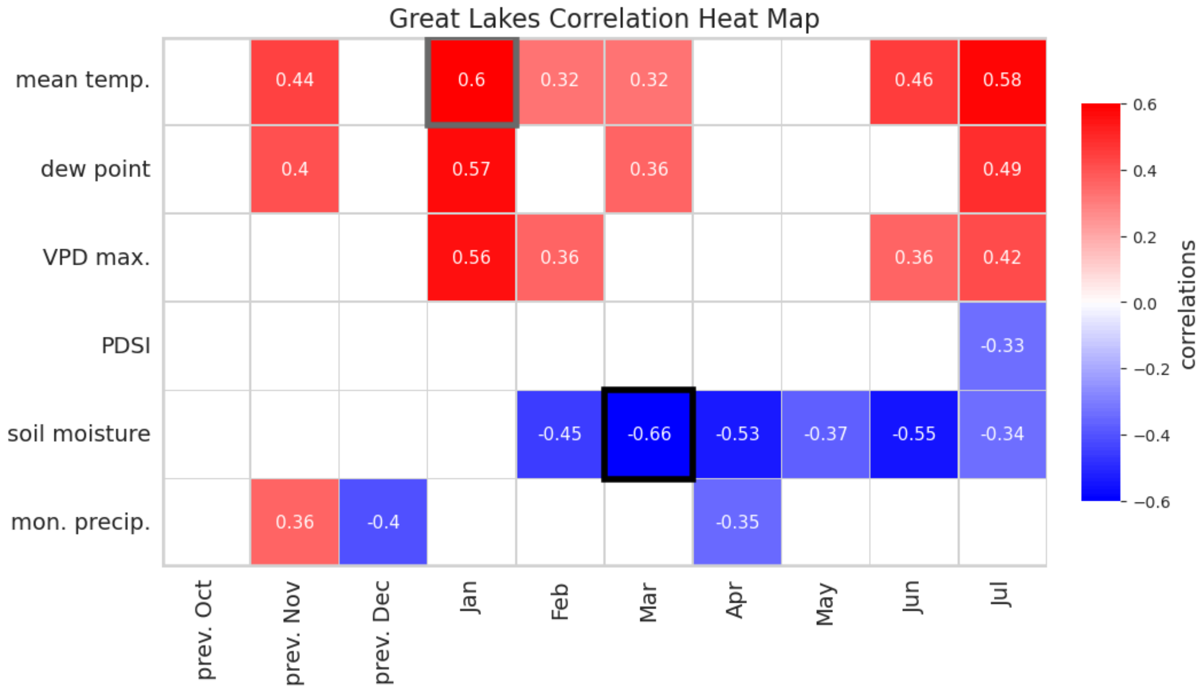

Figure S4: Correlations between Monthly Climate Factors and Annual West Nile Virus Neuroinvasive Disease in the Great Lakes NEON Region. Same as Figure S1 but for the Great Lakes NEON region.

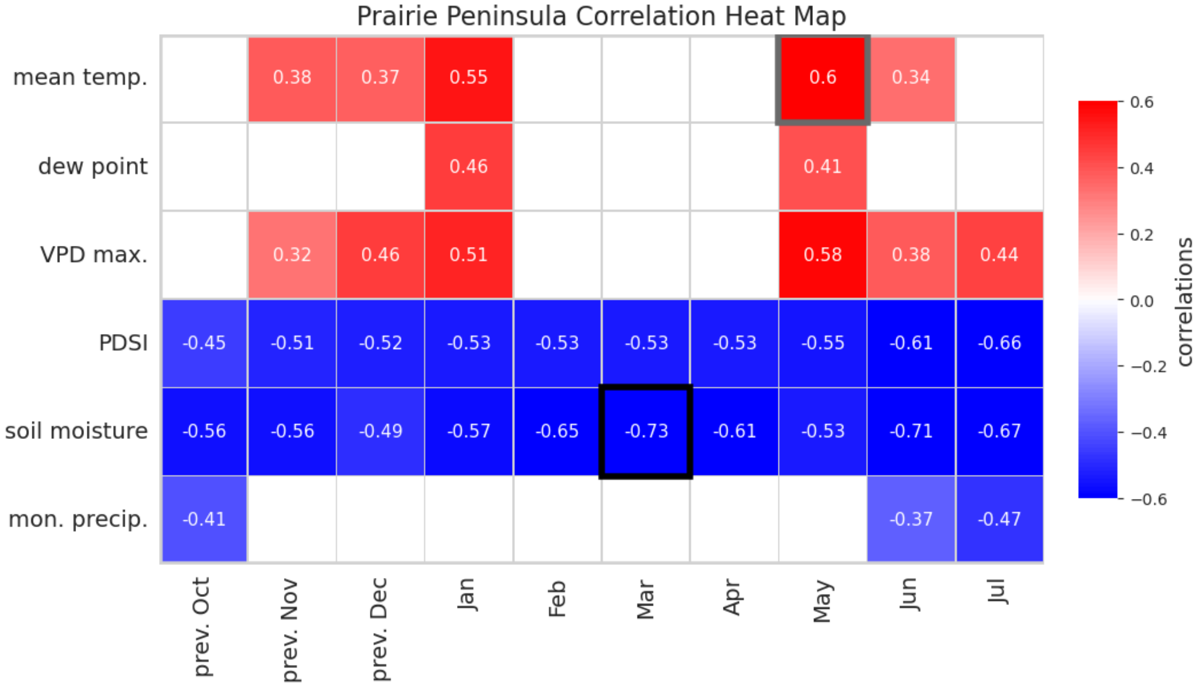

Figure S5: Correlations between Monthly Climate Factors and Annual West Nile Virus Neuroinvasive Disease in the Prairie Peninsula NEON Region. Same as Figure S1 but for the Prairie Peninsula NEON region.

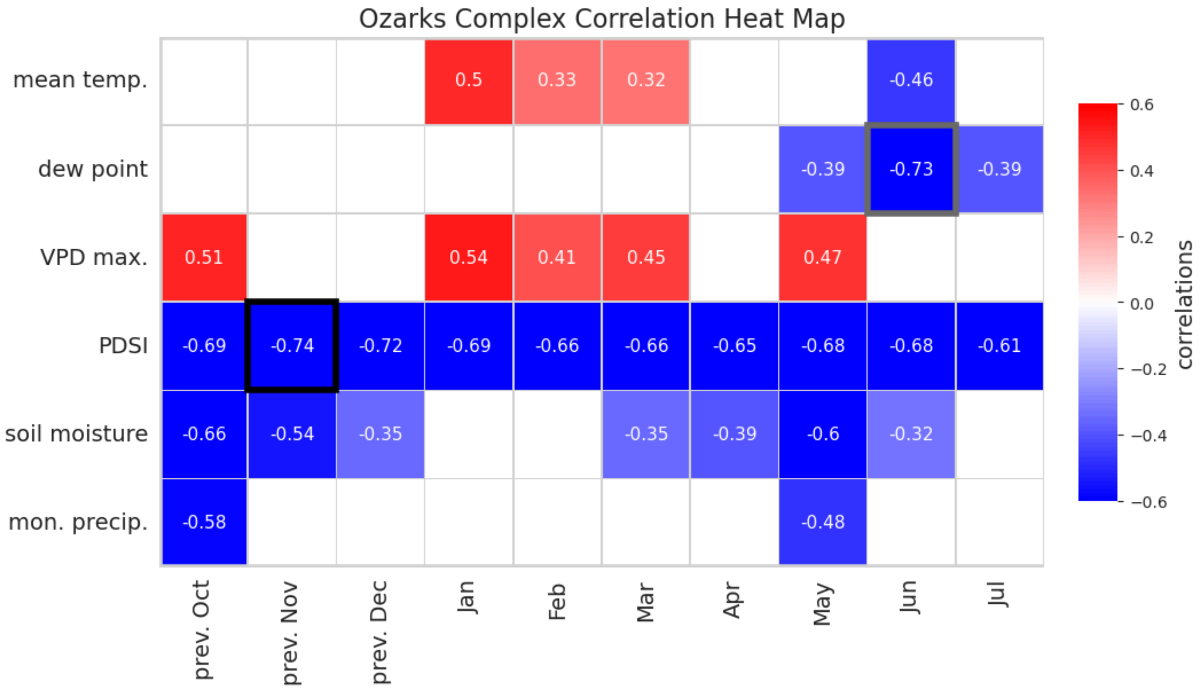

Figure S6: Correlations between Monthly Climate Factors and Annual West Nile Virus Neuroinvasive Disease in the Ozarks Complex NEON Region. Same as Figure S1 but for the Ozarks Complex NEON region.

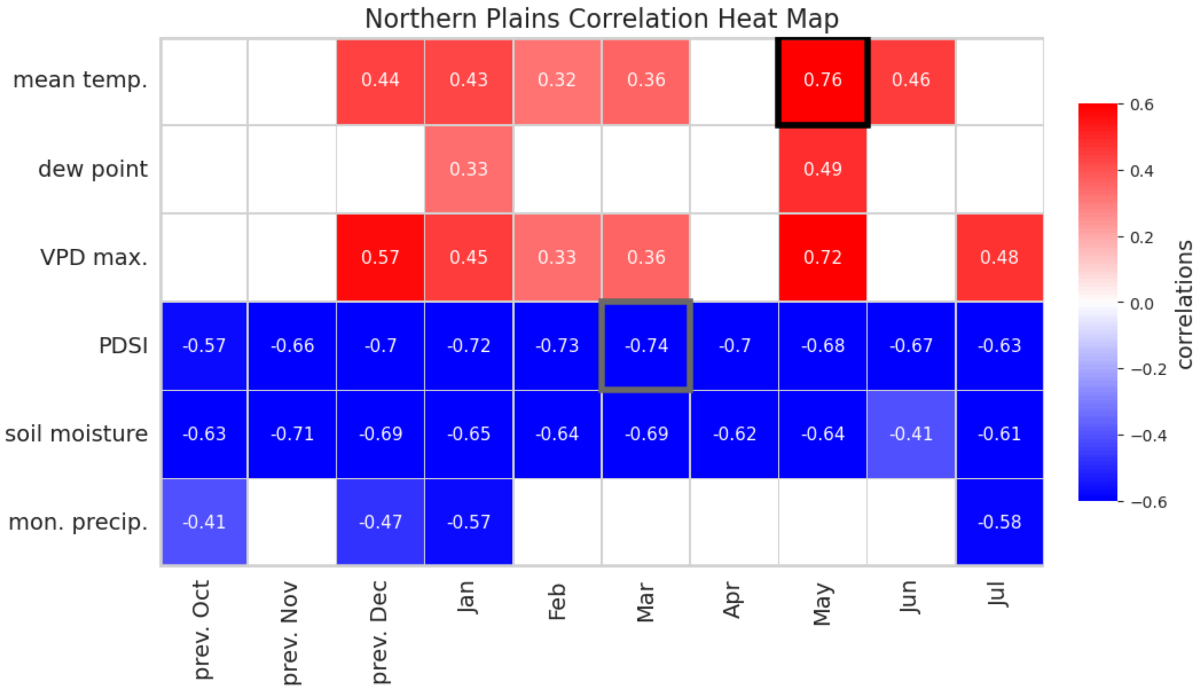

Figure S7: Correlations between Monthly Climate Factors and Annual West Nile Virus Neuroinvasive Disease in the Northern Plains NEON Region. Same as Figure S1 but for the Northern Plains NEON region.

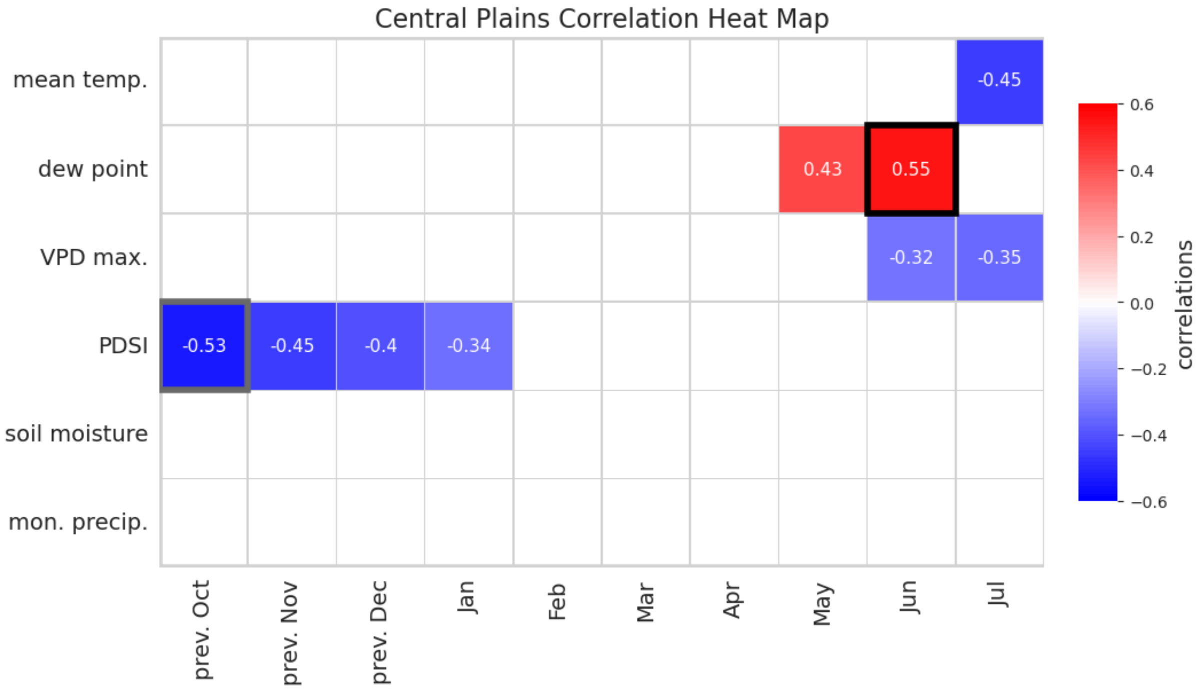

Figure S8: Correlations between Monthly Climate Factors and Annual West Nile Virus Neuroinvasive Disease in the Central Plains NEON Region. Same as Figure S1 but for the Central Plains NEON region.

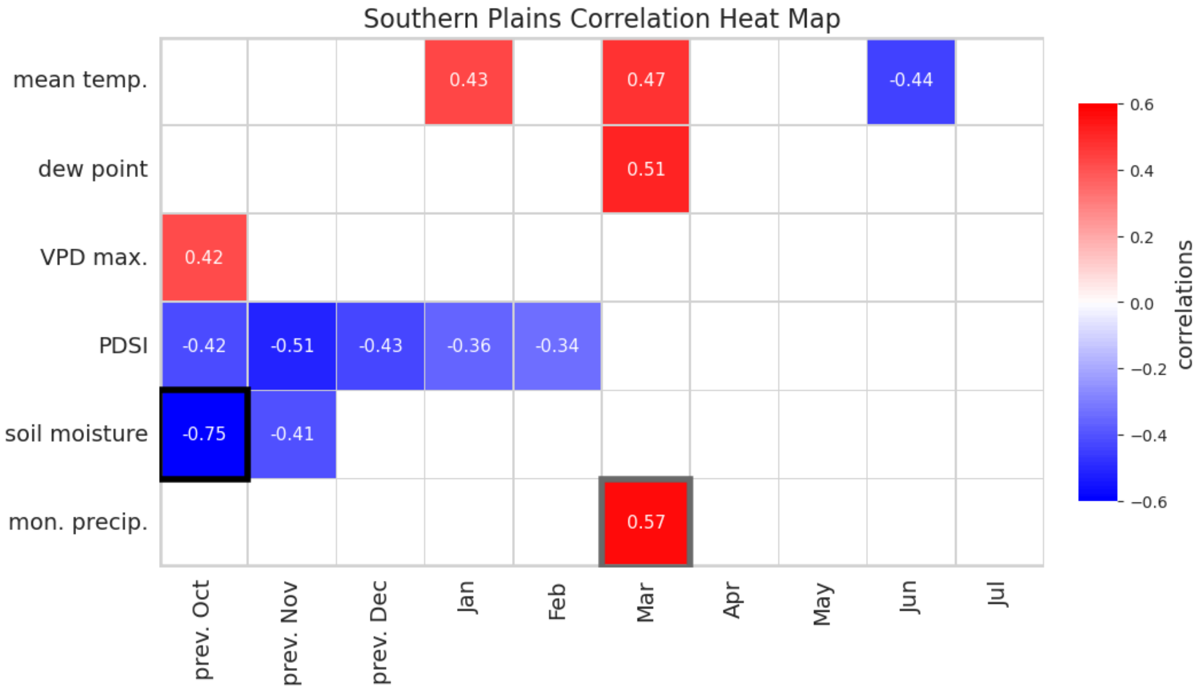

Figure S9: Correlations between Monthly Climate Factors and Annual West Nile Virus Neuroinvasive Disease in the Southern Plains NEON Region. Same as Figure S1 but for the Southern Plains NEON region.

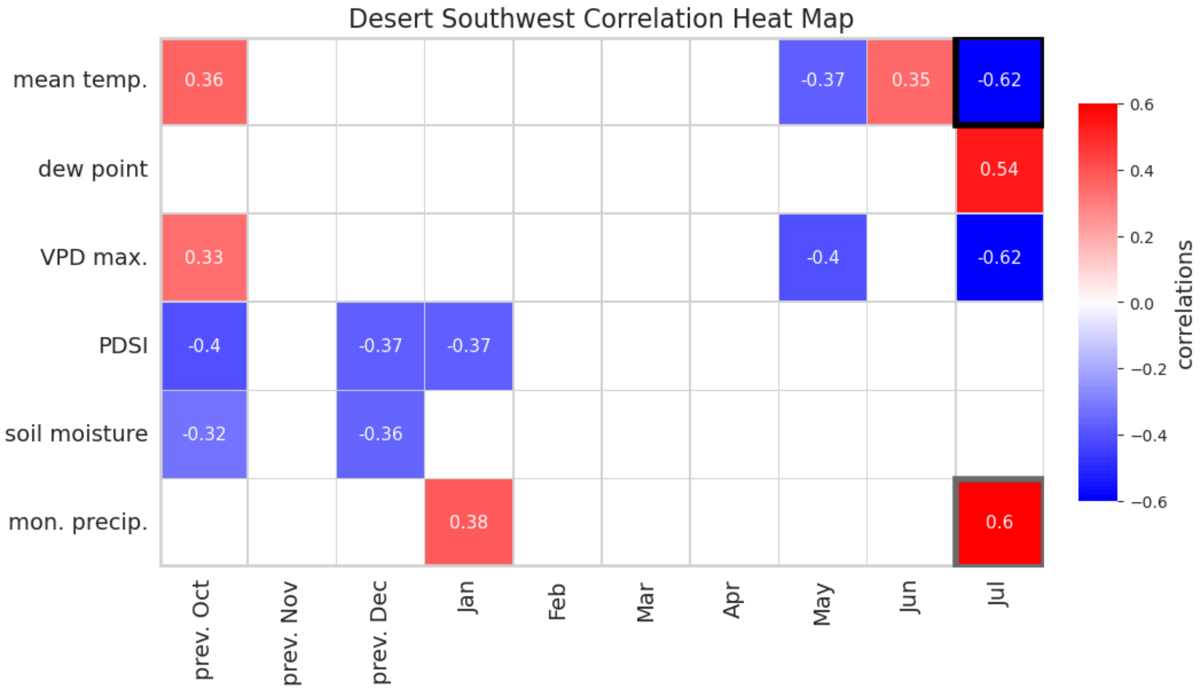

Figure S10: Correlations between Monthly Climate Factors and Annual West Nile Virus Neuroinvasive Disease in the Desert Southwest NEON Region. Same as Figure S1 but for the Desert Southwest NEON region.

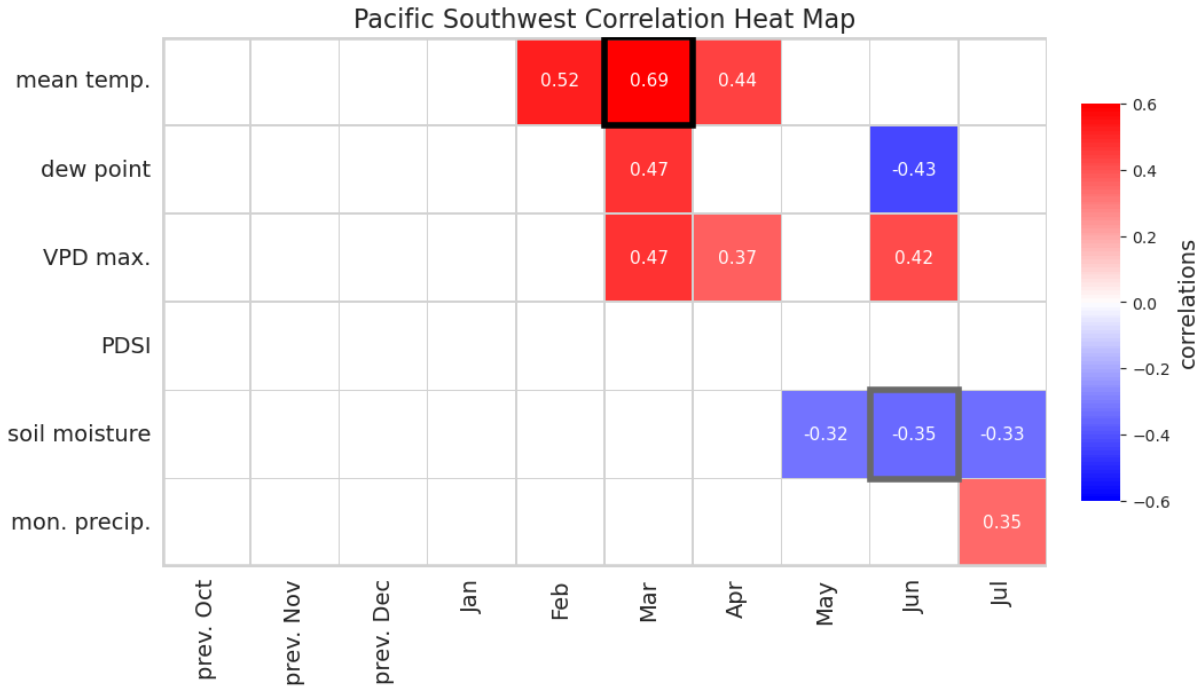

Figure S11: Correlations between Monthly Climate Factors and Annual West Nile Virus Neuroinvasive Disease in the Pacific Southwest NEON Region. Same as Figure S1 but for the Pacific Southwest NEON region.

Table S1: Univariable Bayesian Regression Coefficients. The mean, standard deviation, median, and 95% credible interval bounds are shown for the regional Bayesian regression coefficients between annual WNND and the primary climate variable for the univariable forecast model. Values shown are the mean values of the statistics of the annually generated regressions coefficients from 2005–2022.

**NEON Region**	**Climate Variable**	**Mean**	**Standard Deviation**	**2.5% Credible Interval**	**Median**	**97.5% Credible Interval**
Pacific Southwest	March mean temperature	0.271	0.087	0.097	0.271	0.444
Desert Southwest	July mean temperature	-0.928	0.302	-1.519	-0.928	-0.329
Northern Plains	May mean temperature	-0.447	0.117	-0.673	-0.449	-0.211
Central Plains	June mean dew point	-0.244	0.109	-0.469	-0.241	-0.036
Southern Plains	previous October soil moisture	-0.289	0.412	-1.062	-0.301	0.55
Prairie Peninsula	March soil moisture	-0.23	0.082	-0.39	-0.231	-0.066
Ozarks Complex	previous November PDSI	-0.237	0.067	-0.37	-0.237	-0.105
Great Lakes	March soil moisture	0.388	0.16	0.072	0.388	0.704
Southeast	January soil moisture	-0.044	0.017	-0.079	-0.044	-0.011
Mid Atlantic	May mean temperature	0.327	0.106	0.117	0.327	0.536
Northeast	previous December soil moisture	-0.048	0.025	-0.096	-0.048	0.002

Table S2: Bivariable Bayesian Regression Coefficients. The mean, standard deviation, median, and 95% credible interval bounds are shown for the regional Bayesian regression coefficients between annual WNND and both the primary (top) and secondary (bottom) climate variables for the bivariable forecast model. Values shown are the mean values of the statistics of the annually generated regressions coefficients from 2005–2022.

NEON Region	Climate Variable	Mean	Standard Deviation	2.5% Credible Interval	Median	97.5% Credible Interval
Pacific Southwest	March mean temperature	0.258	0.102	0.055	0.258	0.456
	June soil moisture	-0.002	0.008	-0.018	-0.003	0.014
Desert Southwest	July mean temperature	-0.42	0.415	-1.265	-0.412	0.383
	July precipitation	0.023	0.015	-0.005	0.023	0.052
Northern Plains	May mean temperature	-0.331	0.09	-0.508	-0.332	-0.153
	March PDSI	0.418	0.119	0.188	0.415	0.66
Central Plains	June mean dew point	-0.271	0.098	-0.473	-0.268	-0.083
	previous October PDSI	0.218	0.108	0.008	0.218	0.429
Southern Plains	previous October soil moisture	-0.034	0.01	-0.053	-0.034	-0.014
	March precipitation	0.007	0.008	-0.008	0.007	0.022
Prairie Peninsula	March soil moisture	-0.133	0.086	-0.308	-0.132	0.035
	May mean temperature	0.227	0.117	0.009	0.222	0.475

Ozarks Complex	previous November PDSI	-0.139	0.083	-0.301	-0.139	0.026
	June mean dew point	-0.316	0.173	-0.668	-0.313	0.02
Great Lakes	March soil moisture	0.339	0.15	0.045	0.34	0.634
	January mean temperature	-0.019	0.01	-0.038	-0.019	0.001
Southeast	January soil moisture	-0.038	0.019	-0.074	-0.038	-0.001
	May vapor pressure deficit maximum	0.123	0.098	-0.074	0.123	0.316
Mid Atlantic	May mean temperature	0.366	0.102	0.17	0.365	0.57
	July precipitation	0.012	0.005	0.002	0.012	0.023
Northeast	previous December soil moisture	-0.02	0.02	-0.06	-0.019	0.019
	May mean temperature	0.216	0.117	-0.018	0.217	0.446

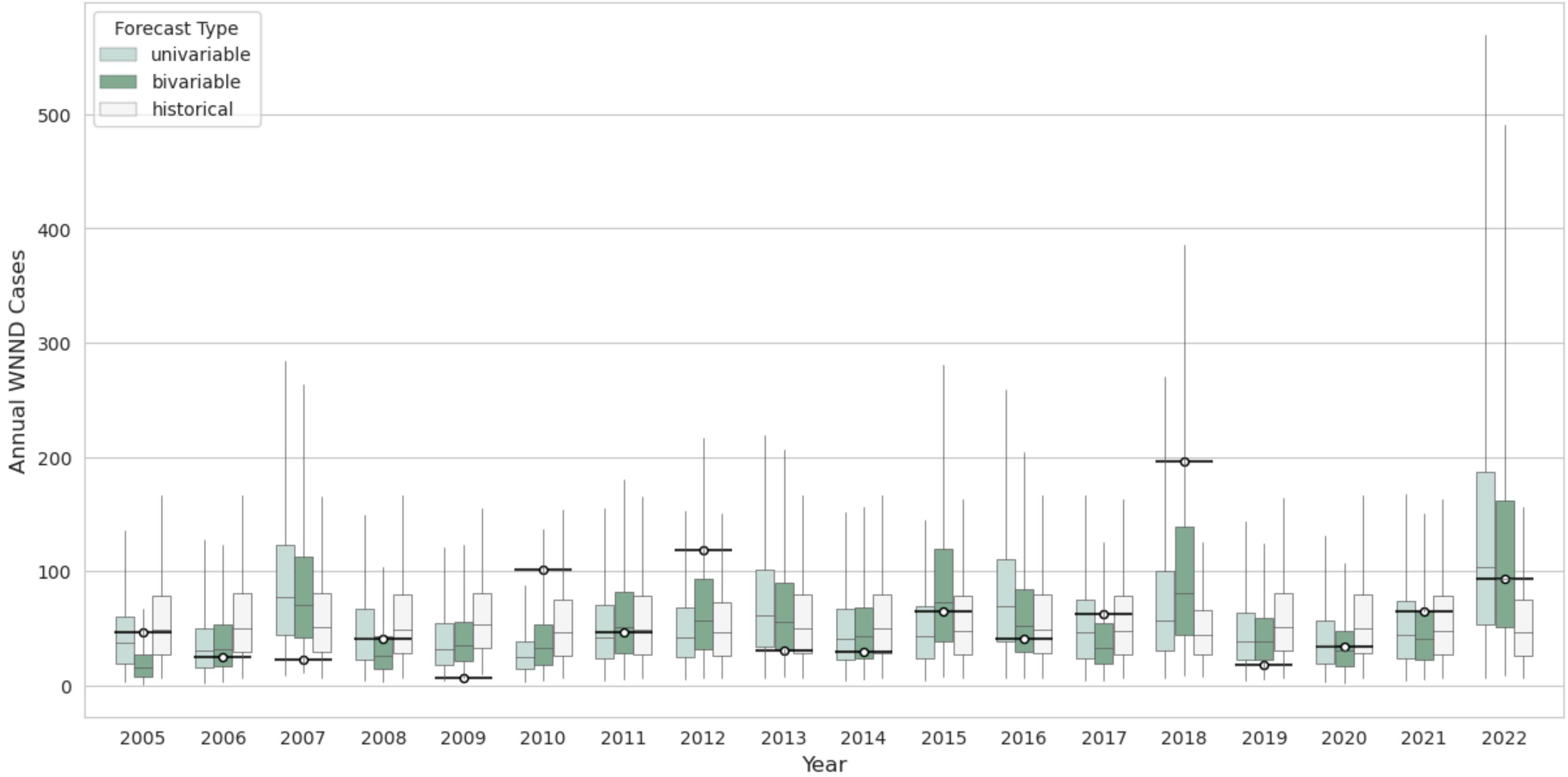

Figure S12: Annual Forecasts and Observations of WNND for the Northeast Region. Box plots of WNND case forecasts for the univariable (light green), bivariable (dark green), and historical benchmark (light grey) models for the Northeast region for each year from 2005–2022. Box encompasses interquartile range and whiskers depict 95% credible intervals. Reported WNND cases for each year are depicted as a circle with horizontal line.

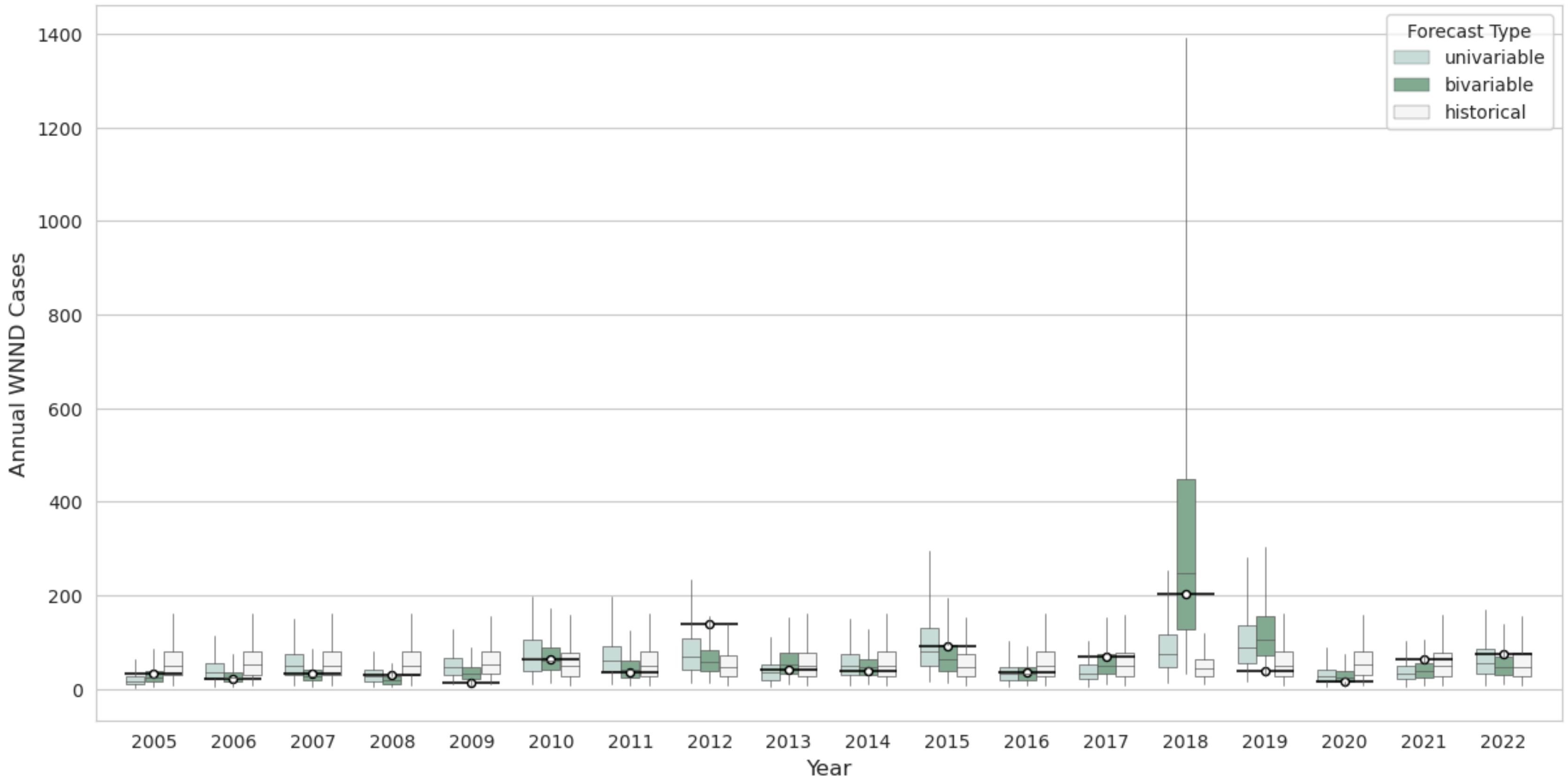

Figure S13: Annual Forecasts and Observations of WNND for the Mid Atlantic Region. Same as Figure S12 but for the Mid Atlantic region.

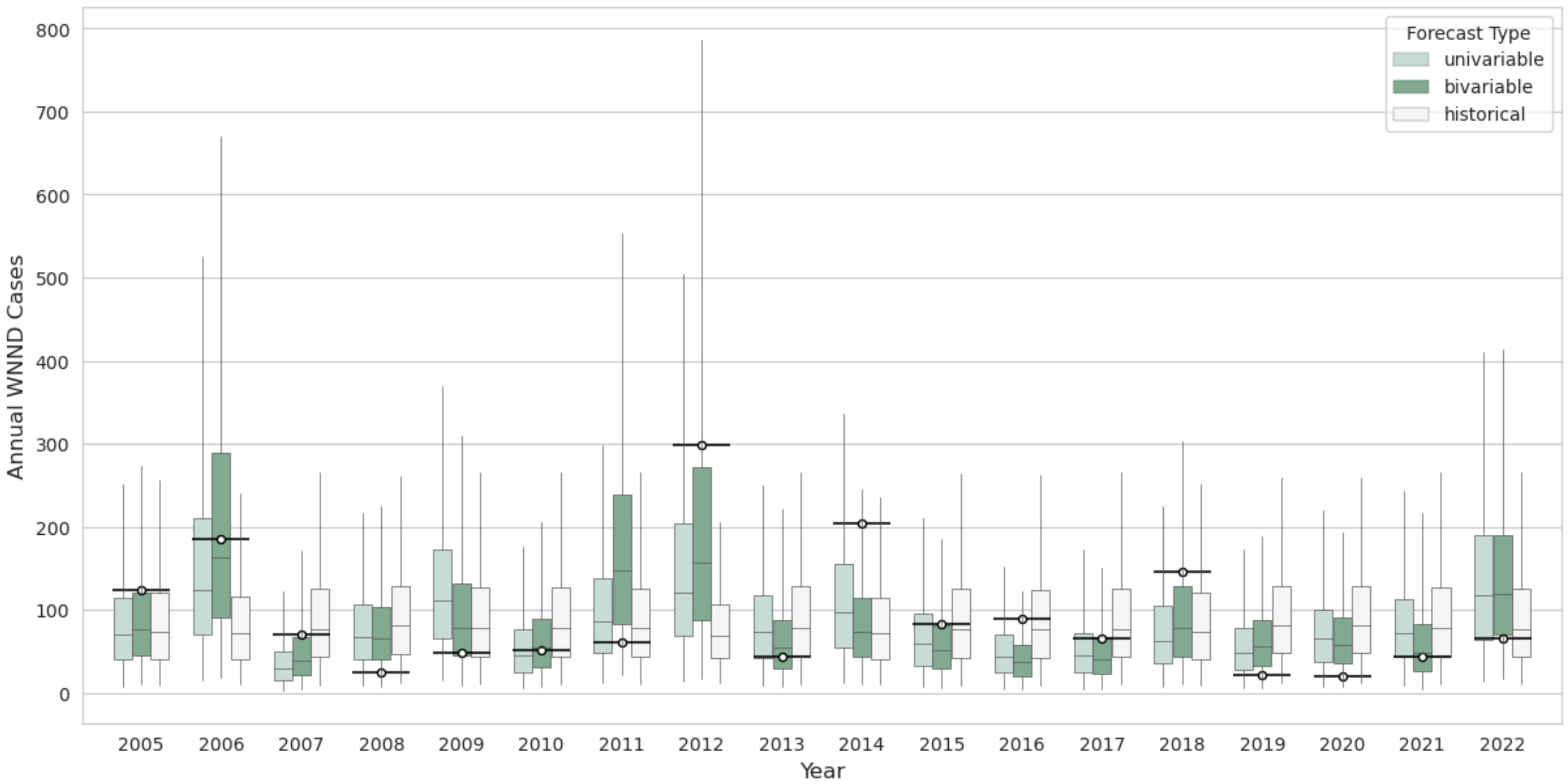

Figure S14: Annual Forecasts and Observations of WNND for the Southeast Region. Same as Figure S12 but for the Southeast region.

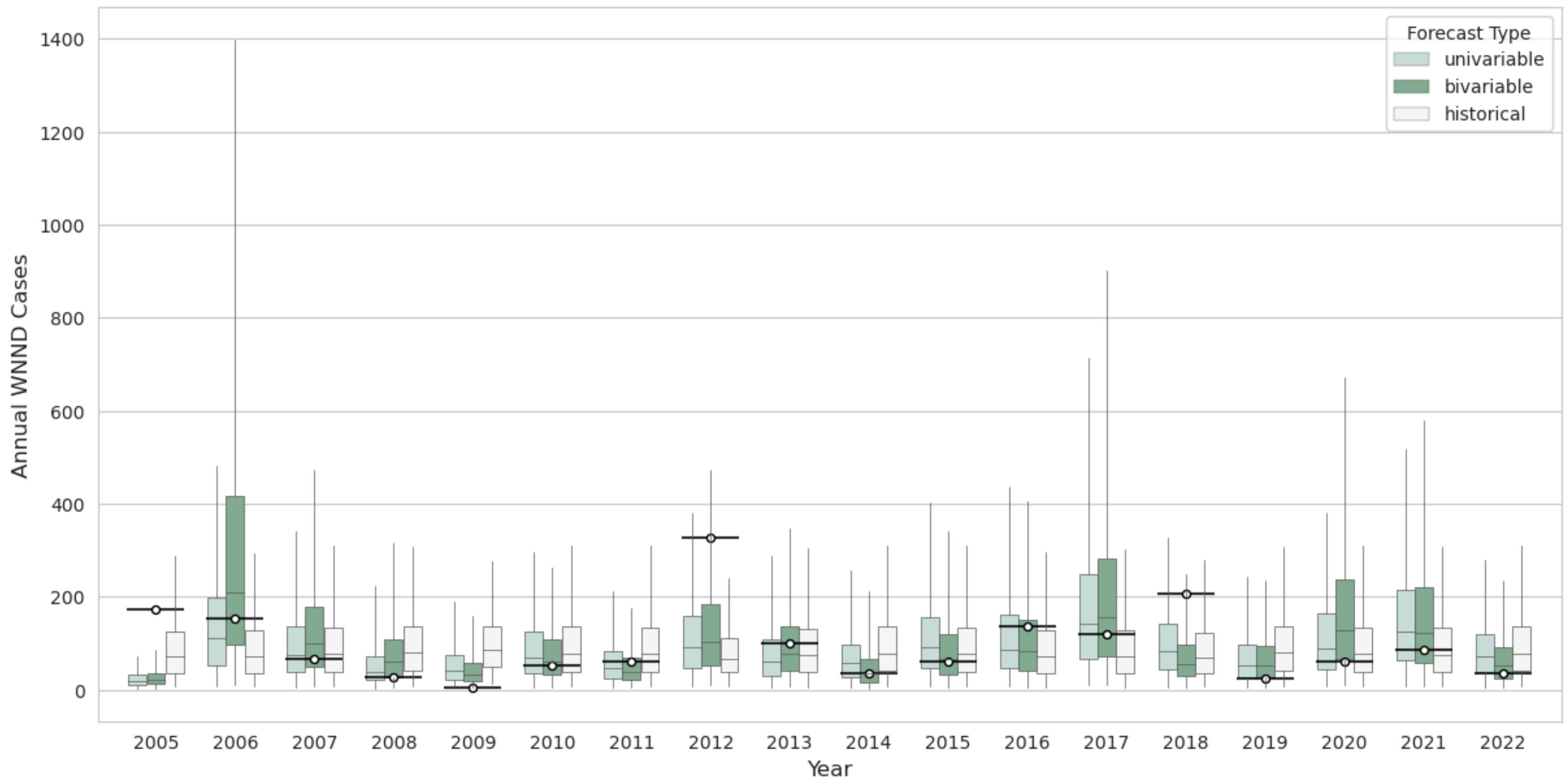

Figure S15: Annual Forecasts and Observations of WNND for the Great Lakes Region. Same as Figure S12 but for the Great Lakes region.

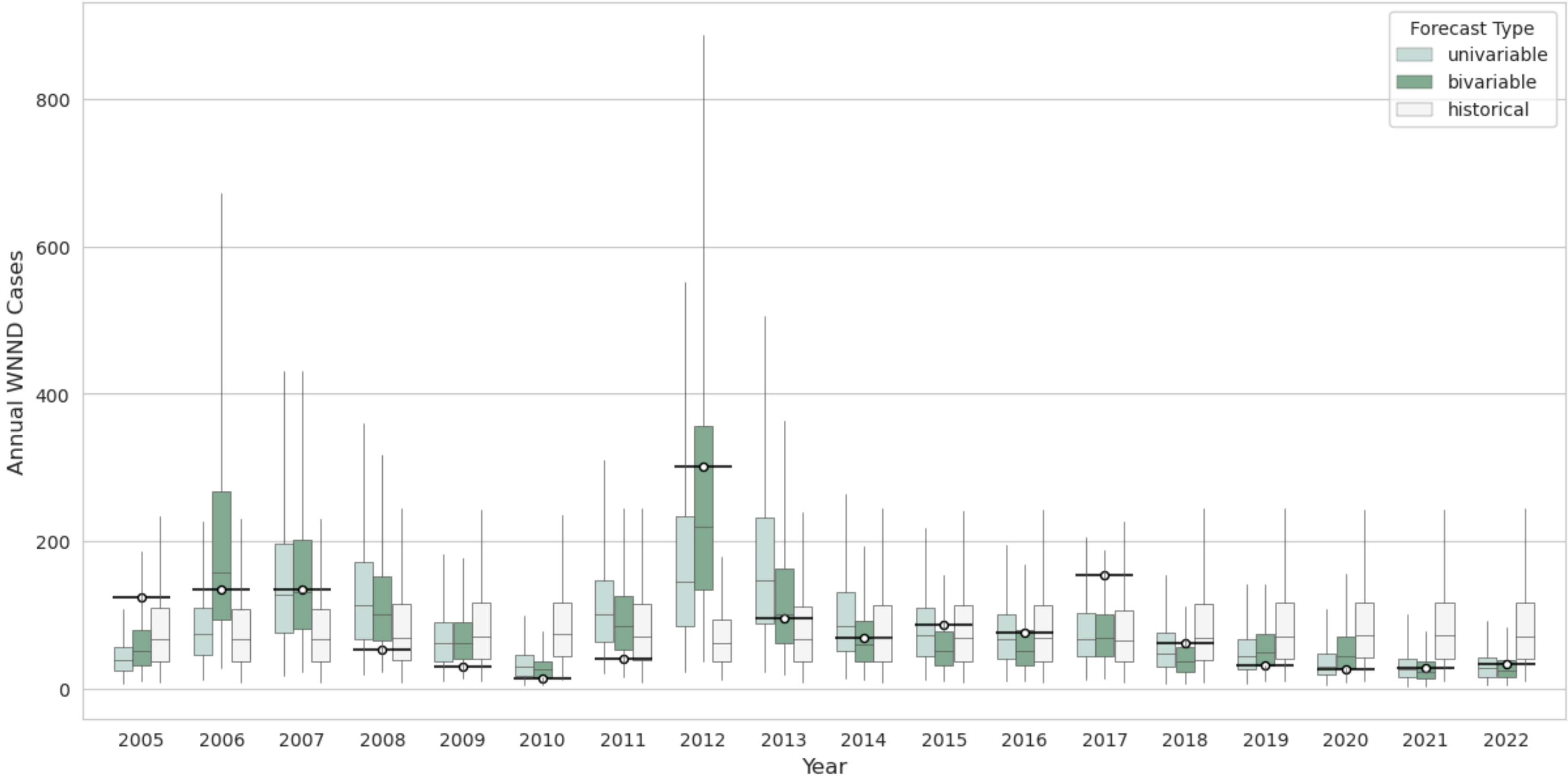

Figure S16: Annual Forecasts and Observations of WNND for the Ozarks Complex Region. Same as Figure S12 but for the Ozarks Complex region.

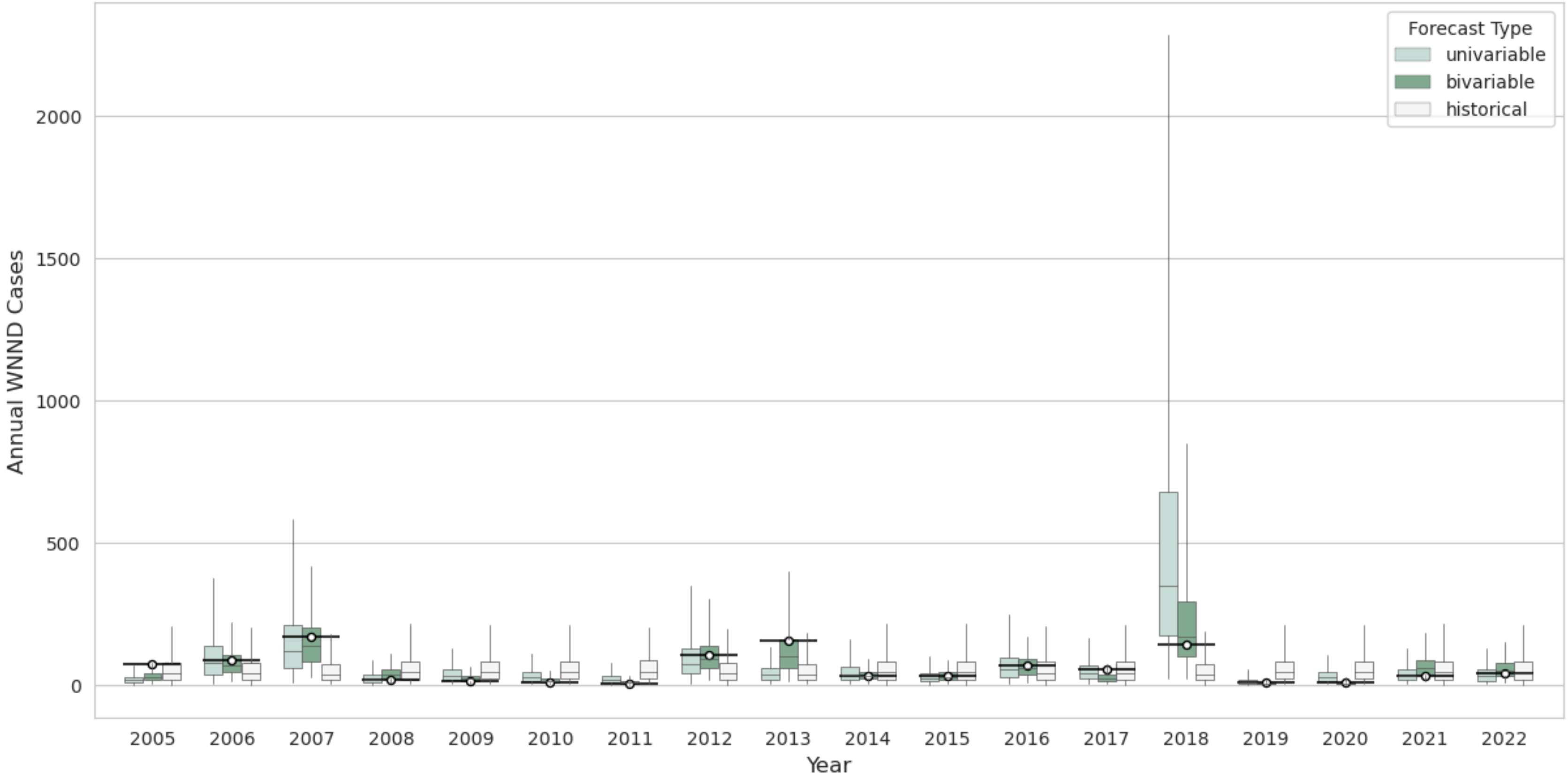

Figure S17: Annual Forecasts and Observations of WNND for the Northern Plains Region. Same as Figure S12 but for the Northern Plains region.

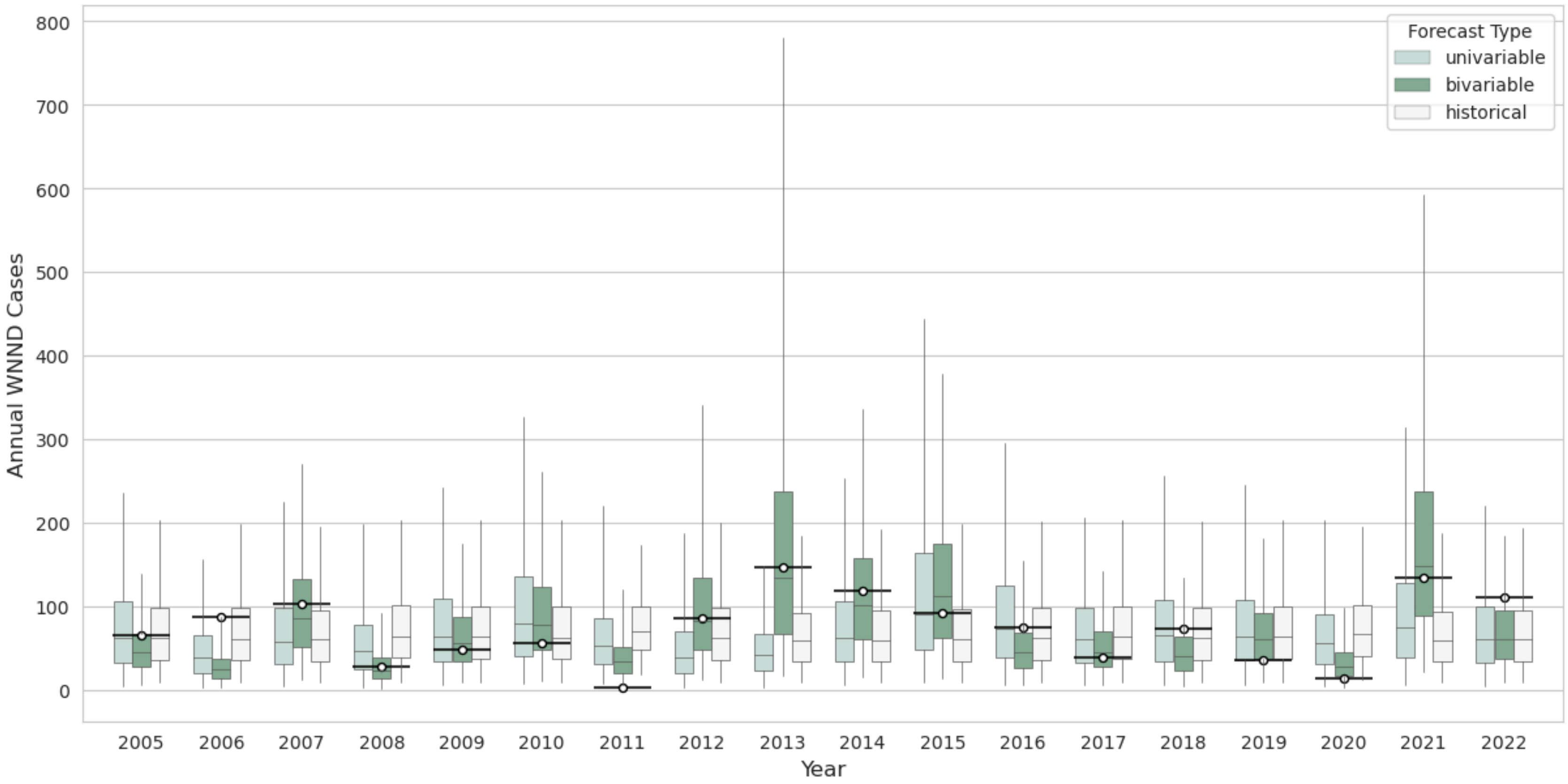

Figure S18: Annual Forecasts and Observations of WNND for the Central Plains Region. Same as Figure S12 but for the Central Plains region.

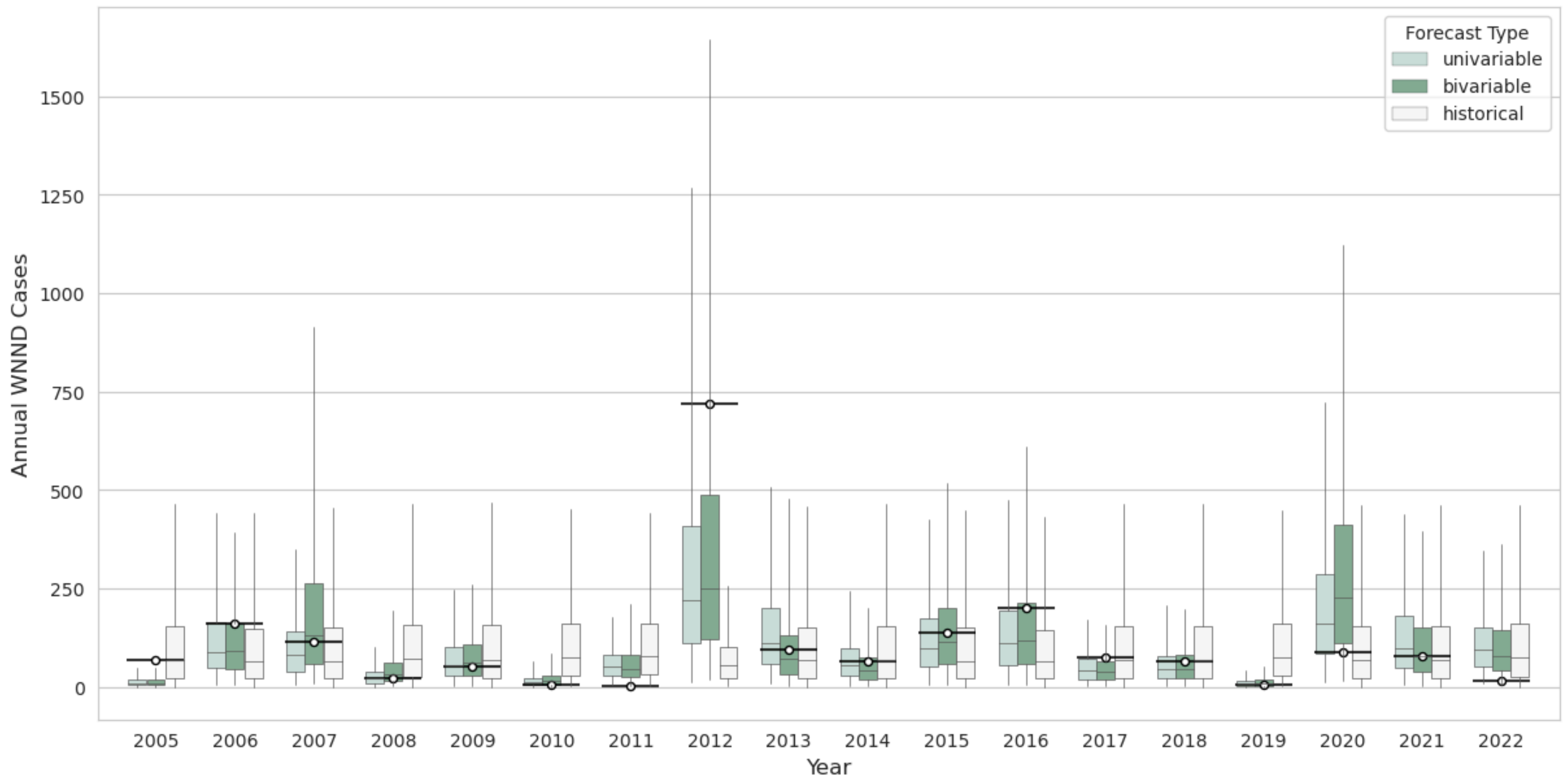

Figure S19: Annual Forecasts and Observations of WNND for the Southern Plains Region. Same as Figure S12 but for the Southern Plains region.

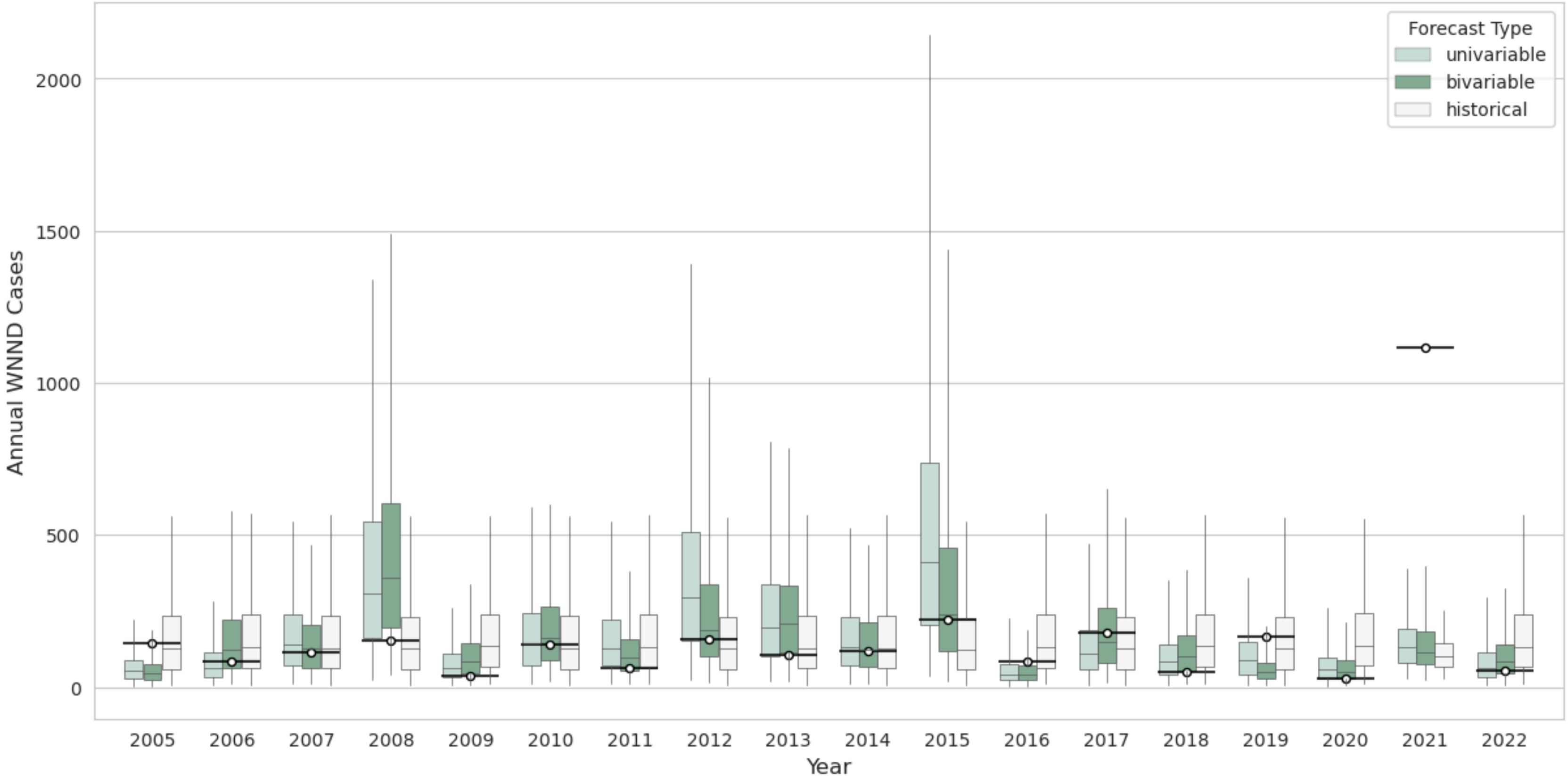

Figure S20: Annual Forecasts and Observations of WNND for the Desert Southwest Region. Same as Figure S12 but for the Desert Southwest region.

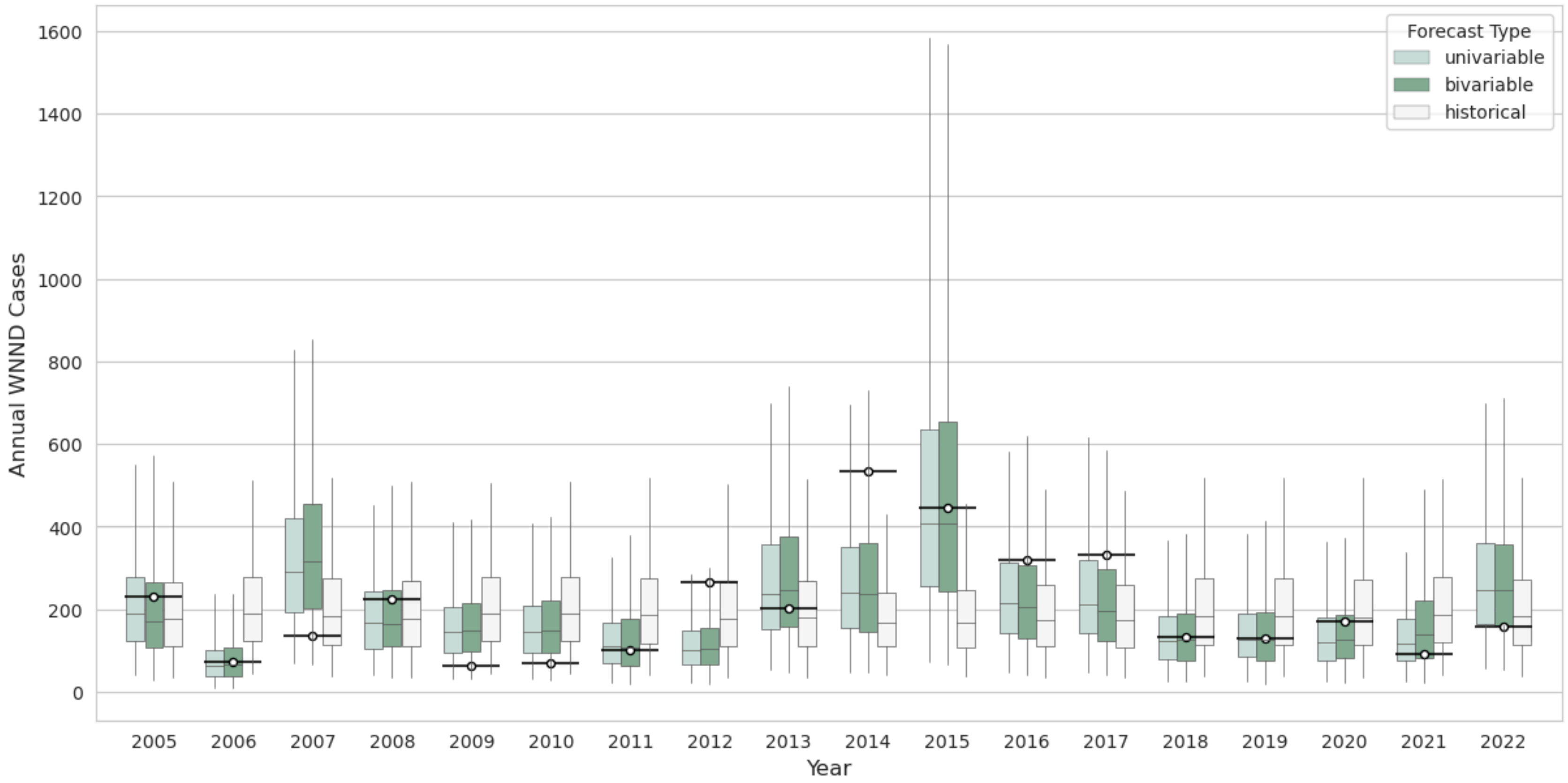

Figure S21: Annual Forecasts and Observations of WNND for the Pacific Southwest Region. Same as Figure S12 but for the Pacific Southwest region.

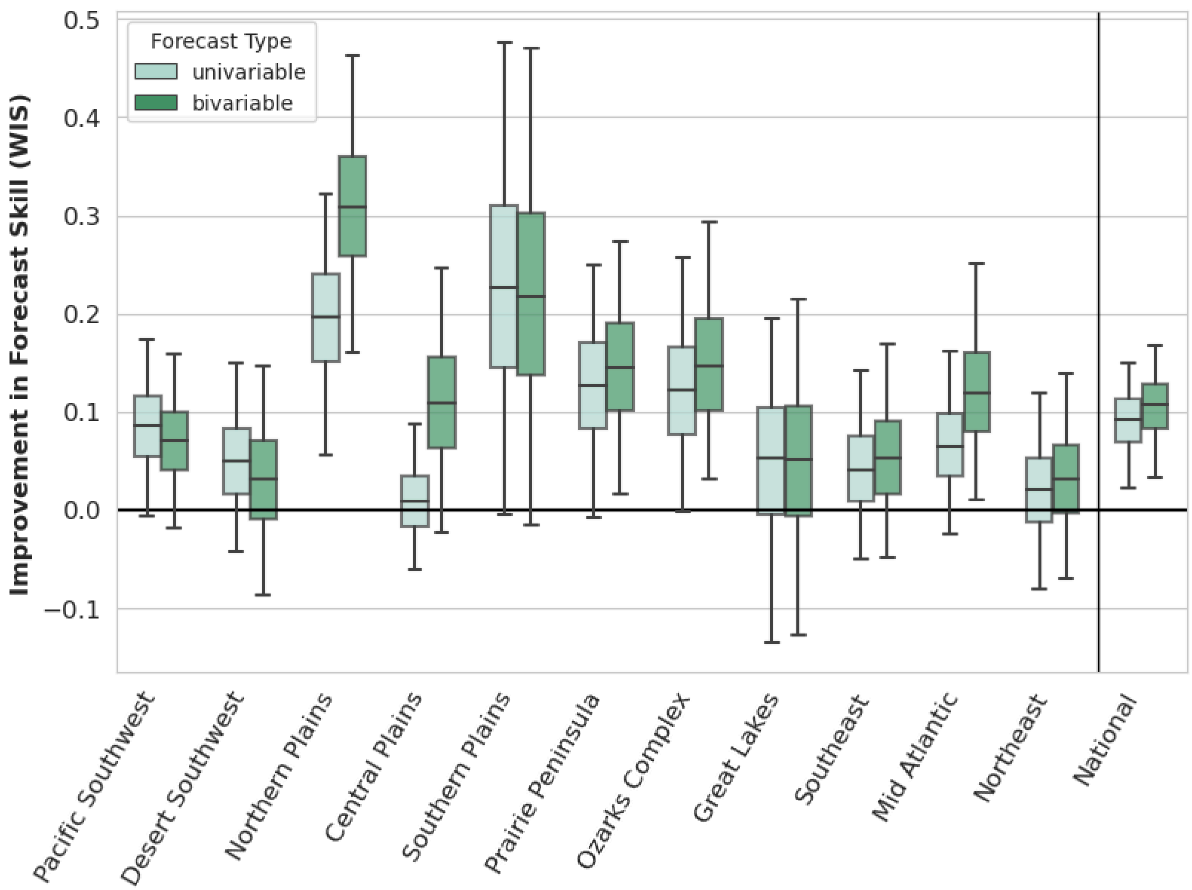

Figure S22: Weighted Interval Score Improvement in Regional Forecast Skill of Climate-Informed Forecast Models Compared to Historical Negative Binomial Benchmark. Box plots of regional and nationally aggregated differences in retrospective forecast skill between univariable (light green) and bivariable (dark green) climate-informed forecast models and historical negative binomial distribution for 2005–2022. Score improvements are calculated as the climate-informed forecast model weighted interval scores less the historical negative binomial weighted interval scores. These differences are then inverted so that above zero indicate regions where forecast skill is better in the climate-informed forecast model. Box encompasses interquartile range and whiskers depict 95% confidence intervals as determined by bootstrapping. NEON regions are listed from West to East.

Table S3: Comparison of Multi-Level County-Specific Climate-Informed WNV Forecast with 2022 CDC WNV Forecasting Challenge Forecast Models.

Comparison Model	Improvement in Mean Score (%)	p-value
ensemble	1.0	n.s.
historical negative binomial	2.3	0.011
hybrid-hybrid	3.8	0.001
MSSM-WED	5.6	<0.001
LANL-NBandP	8.8	<0.001
Datart-PoissonFE	17.1	<0.001
AMbeRland-RandomForest_anomaly	28.9	<0.001
FINforWN-MCMaWN	33.3	<0.001
CDC-NaiveHist	34.4	<0.001
USC-INLA	44.0	<0.001
kansas-bayesian	51.2	<0.001

Mean score differences and associated one-sided p-values for improvement of county-level climate-informed WNV forecast compared to each model analyzed within the 2022 CDC WNV Forecasting Challenge.

Table S4: Improvements in Weighted Interval Score Components in the Univariable Climate-Informed Retrospective Forecast over the Historical Negative Binomial Model. Regional mean weighted interval scores from 2005-2022 for retrospective forecasts of the historical negative binomial model and the univariable climate-informed model, along with differences in total mean weighted interval score and the dispersion, overprediction, and underprediction components. Differences are computed as the historical negative binomial score minus the corresponding univariable climate-informed score so that improvements in weighted interval score components in the univariable model compared to the historical negative binomial baseline are positive.

NEON Region	Historical Negative Binomial Total WIS	Univariable Climate-Informed Forecast Total WIS	Improvement in Total WIS	Improvement in Dispersion	Improvement in Overprediction	Improvement in Underprediction
Pacific Southwest	0.34	0.26	0.08	0.00	0.05	0.04
Desert Southwest	0.44	0.39	0.05	0.02	0.05	-0.02
Northern Plains	0.59	0.40	0.19	0.04	0.11	0.04
Central Plains	0.43	0.42	0.01	-0.03	0.05	-0.02
Southern Plains	0.7	0.47	0.23	0.08	0.11	0.03
Prairie Peninsula	0.44	0.31	0.13	0.02	0.08	0.03
Ozarks Complex	0.42	0.30	0.12	0.02	0.07	0.03
Great Lakes	0.51	0.46	0.05	-0.01	0.08	-0.03
Southeast	0.41	0.36	0.05	0.00	0.03	0.02
Mid Atlantic	0.38	0.31	0.07	0.01	0.03	0.03
Northeast	0.4	0.38	0.02	-0.01	0.04	0.00
Average	0.46	0.37	0.09	0.01	0.06	0.01

Table S5: Improvements in Weighted Interval Score Components in the Bivariable Climate-Informed Retrospective Forecast over the Historical Negative Binomial Model. Regional mean weighted interval scores from 2005-2022 for retrospective forecasts of the historical negative binomial model and the bivariable climate-informed model, along with differences in total mean weighted interval score and the dispersion, overprediction, and underprediction components. Differences are computed as the historical negative binomial score minus the corresponding univariable climate-informed score so that improvements in weighted interval score components in the bivariable model compared to the historical negative binomial baseline are positive.

NEON Region	Historical Negative Binomial Total WIS	Bivariable Climate-Informed Forecast Total WIS	Improvement in Total WIS	Improvement in Dispersion	Improvement in Overprediction	Improvement in Underprediction
Pacific Southwest	0.34	0.27	0.07	-0.01	0.04	0.04
Desert Southwest	0.44	0.41	0.03	0.03	0.05	-0.04
Northern Plains	0.59	0.28	0.31	0.09	0.15	0.07
Central Plains	0.43	0.32	0.11	0.00	0.10	0.01
Southern Plains	0.7	0.48	0.22	0.07	0.11	0.03
Prairie Peninsula	0.44	0.29	0.15	0.02	0.09	0.04
Ozarks Complex	0.42	0.27	0.15	0.03	0.08	0.04
Great Lakes	0.51	0.46	0.05	-0.01	0.09	-0.03
Southeast	0.41	0.35	0.06	-0.01	0.03	0.03
Mid Atlantic	0.38	0.25	0.13	0.02	0.05	0.05
Northeast	0.4	0.37	0.03	-0.01	0.04	0.01
Average	0.46	0.34	0.12	0.02	0.07	0.02

Table S6: Pairwise Comparison of Forecast Skill. Regional comparison of forecast skill between the retrospective forecasts for the univariable model, bivariable model, and historical negative binomial baseline. Values shown are the percentage of paired bootstrapped comparisons in which one model in the described pairing outperformed the other (model pairings described in column headers).

NEON Region	Likelihood of Univariable Forecast Outperforming Historical Benchmark (%)	Likelihood of Bivariable Forecast Outperforming Historical Benchmark (%)	Likelihood of Bivariable Forecast Outperforming Univariable Forecast (%)
Pacific Southwest	96.81	93.74	0.29
Desert Southwest	85.04	70.51	24.95
Northern Plains	99.62	99.98	98.82
Central Plains	58.14	94.68	98.09
Southern Plains	97.19	96.72	31.55
Prairie Peninsula	96.92	98.56	75.95
Ozarks Complex	97.37	99.49	86.33
Great Lakes	73.17	72.64	54.65
Southeast	80.63	83.58	66.96
Mid Atlantic	92.01	98.37	95.82
Northeast	66.08	72.58	64.61
National Aggregate	99.09	99.81	98.7

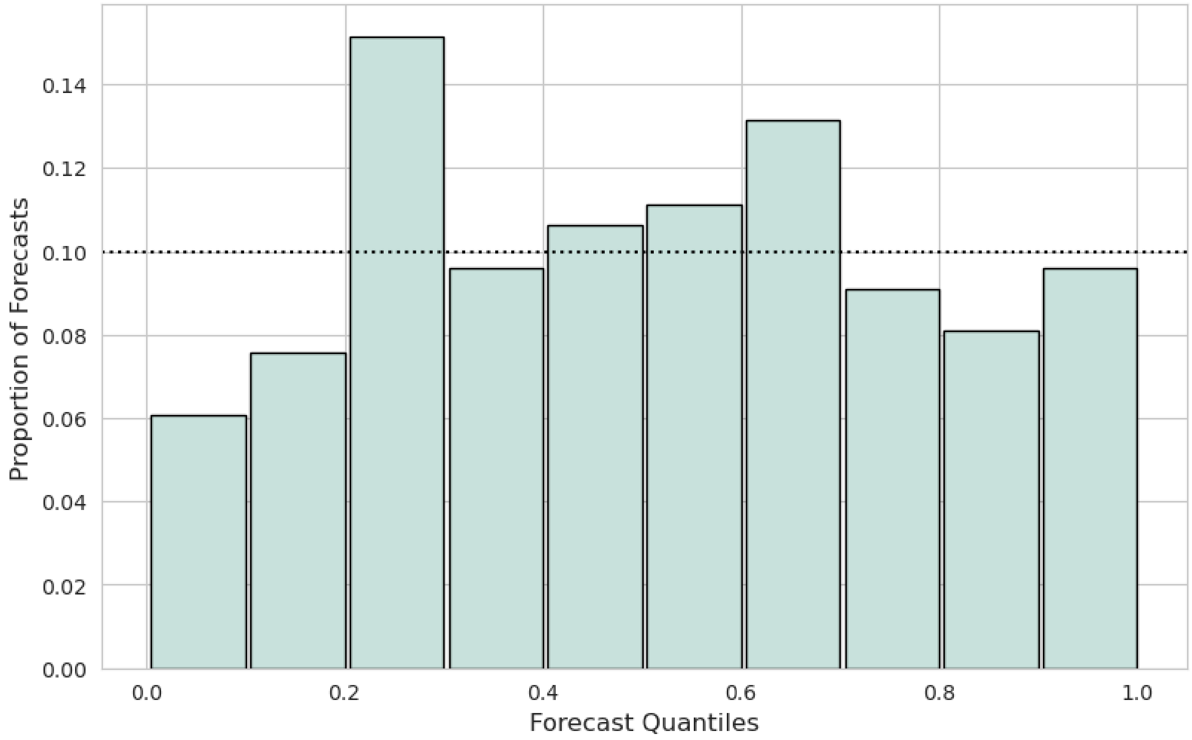

Figure S23: Probability Integral Transform Histogram for Retrospective Forecasts of the Univariable Model. Bars represent the proportion of region-year retrospective forecasts (n = 198) for which the reported WNND cases were within a given quantile bin. The dashed horizontal line represents the proportion of a perfectly calibrated forecast (0.10).

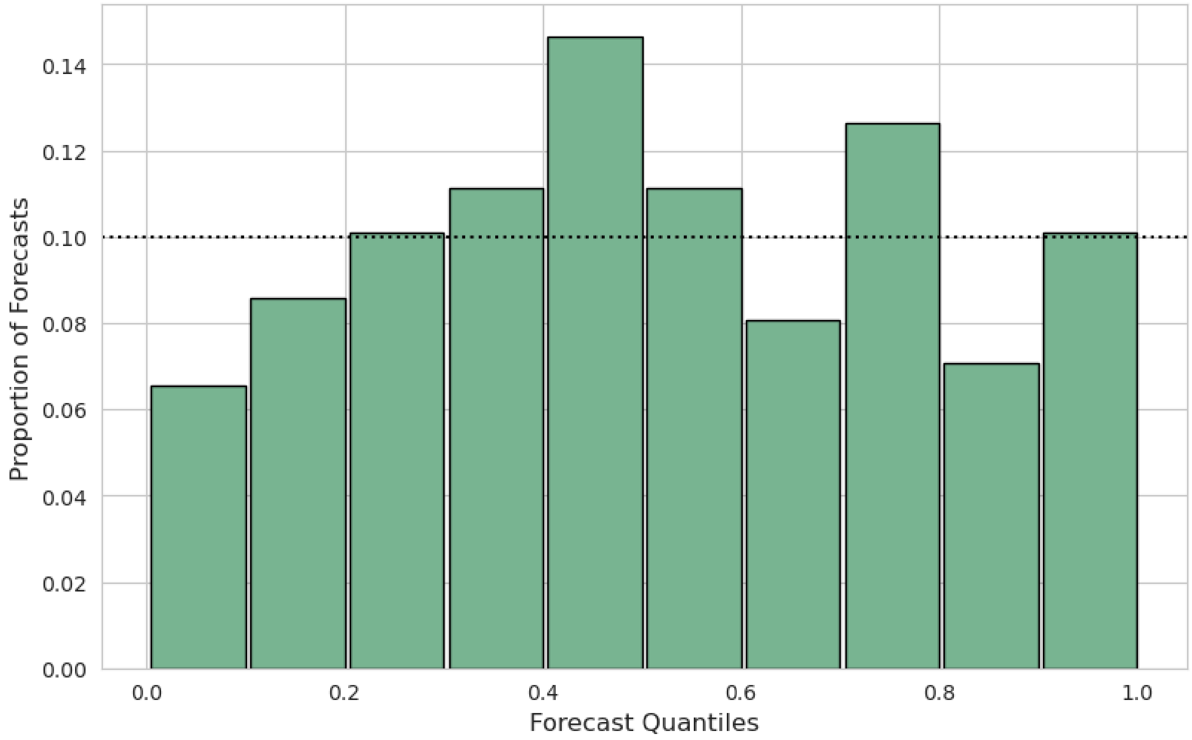

Figure S24: Probability Integral Transform Histogram for Retrospective Forecasts of the Bivariable Model. Bars represent the proportion of region-year retrospective forecasts (n = 198) for which the reported WNND cases were within a given quantile bin. The dashed horizontal line represents the proportion of a perfectly calibrated forecast (0.10).

Table S7: Region-Specific Interval Coverage Statistics for the Univariable and Bivariable Forecasts. Proportion of annual reported WNND cases occurring within the middle 50% and 90% of forecast distributions for each region-year retrospective forecast for both the univariable and bivariable models.

NEON Region	Univariable 50% Interval Coverage (%)	Univariable 90% Interval Coverage (%)	Bivariable 50% Interval Coverage (%)	Bivariable 90% Interval Coverage (%)
Pacific Southwest	56	94	56	94
Desert Southwest	61	94	50	89
Northern Plains	61	89	61	94
Central Plains	50	89	61	89
Southern Plains	67	89	56	89
Prairie Peninsula	56	94	56	100
Ozarks Complex	56	94	50	100
Great Lakes	67	83	67	83
Southeast	44	100	33	94
Mid Atlantic	56	100	67	94
Northeast	61	94	50	94
National Mean	58	93	55	93
